# A Novel Grape-Derived Prebiotic Selectively Enhances Abundance and Metabolic Activity of Butyrate-Producing Bacteria in Faecal Samples

**DOI:** 10.3389/fmicb.2021.639948

**Published:** 2021-03-23

**Authors:** Lia Oliver, Sara Ramió-Pujol, Joan Amoedo, Marta Malagón, Marta Serrano, Anna Bahí, Aleix Lluansí, Leyanira Torrealba, David Busquets, Laura Pardo, Mariona Serra-Pagès, Xavier Aldeguer, Jesús Garcia-Gil

**Affiliations:** ^1^GoodGut SL, Girona, Spain; ^2^Institut d’Investigació Biomèdica de Girona-IDIBGI, Salt, Spain; ^3^Hospital Universitari de Girona Dr. Josep Trueta, Girona, Spain; ^4^Department of Biology, University of Girona, Girona, Spain

**Keywords:** prebiotic, gut microbiota, butyrate, gut health, irritable bowel syndrome, inflammatory bowel disease

## Abstract

Inflammatory bowel disease (IBD) and irritable bowel syndrome (IBS) patients have different faecal microbiota profiles compared to healthy controls. Prebiotics intake influences intestinal microbiota composition which in turn influence the growth of short-chain fatty acids (SCFA) producing bacteria. This study aimed to evaluate the capacity of Previpect, a new prebiotic obtained from grapes fibre, to balance the dysbiosis found in patients with intestinal disorders. This was achieved through the analysis of specific bacterial markers and SCFA production using an *in vitro* fermentation system and comparing the obtained results with those obtained with other commercial prebiotics. Fresh faecal samples from patients with IBD (*N* = 6), IBS (*N* = 3), and control subjects (*N* = 6) were used. Previpect showed high fermentative ability enabling the growth of butyrate producing bacteria and increasing SCFA concentration up to 2.5-fold. Previpect is a promising prebiotic which may be used as a therapeutic strategy towards promotion of intestinal microbiota restoration, microbial healing, and as a preventive supplement for healthy individuals.

## Introduction

In recent years, intestinal health has been increasingly linked to a reduction of the risk of suffering from several chronic diseases. Intestinal health leads to an increased interest in the use of prebiotics as functional food ingredients to improve health (Laparra and Sanz, 2010). Prebiotics aim to stimulate the selective growth of the potentially health-promoting indigenous microorganisms; hence, modulating the composition of the natural ecosystem (Markowiak and Ślizewska, 2017). Besides, dietary prebiotics have the potential advantage of not being susceptible to antibiotics (Terpou et al., 2019). Many food oligosaccharides and polysaccharides, such as dietary fibre, fructooligosaccharides (FOS), inulin, galactooligosaccharides (GOS), and other related carbohydrates, have been reported to show prebiotic properties (Rycroft et al., 2001).

Prebiotics may promote a therapeutic effect in some intestinal diseases through different mechanisms. Numerous studies have revealed that faecal microbiota has a different composition in inflammatory bowel disease (IBD) and irritable bowel syndrome (IBS) patients when compared to healthy controls (Sokol et al., 2006; Kassinen et al., 2007; Salonen et al., 2010; Chassaing and Michaud, 2011), which reveals an overall reduction in biodiversity, especially in the Firmicutes phylum. This phylogenetic group includes several butyrate-producing bacteria, notably *Faecalibacterium prausnitzii*, which is a dominant species in the healthy human gut microbiota (Macfarlane et al., 2009; Seksik, 2010; Ghoshal et al., 2012; Manichanh et al., 2012; Collins, 2014). In IBD patients, differences were observed both between active and non-active stages of the disease and between inflamed and non-inflamed regions of the intestine (Swidsinski et al., 2008). Prebiotic intake influences the composition of intestinal microbiota and alters its metabolic properties by increasing the production of short-chain fatty acids (SCFA). This increase may lower the pH of the colonic environment and, thus, inhibit the growth of potentially pathogenic microorganisms (Orel and Trop, 2014). Among SCFA, butyrate stands out, playing a trophic role as a nutrient for colonocytes and enhancing repair of the injured gut epithelium in IBD. Besides, evidence shows that butyrate acts directly as an anti-inflammatory agent by inactivating the intracellular transcriptional factor NFκB pathway, consequently attenuating the synthesis of inflammatory cytokines (Kanauchi et al., 2005).

A novel prebiotic product denominated “Previpect” is composed of grape by-product from winemaking, specifically originated from white grapes class of *Vitis vinifera* L. This novel prebiotic is obtained by drying the residues of the refuse from grapefruit pressing, removing seeds, and other plant particles and subsequent grinding. Previpect has a high content of insoluble fibre, which makes it an excellent prebiotic candidate.

This study aimed to evaluate the prebiotic properties of Previpect by assessing fermentation profiles such as intestinal microbiota and bacterial SCFA production in an *in vitro* fermentation system. In this experiment, fresh faecal samples from control subjects (CS) and patients suffering from IBD and IBS were used as inocula. Besides, Previpect fermentability was compared with a variant of our prebiotic, which is produced following the same procedure as Previpect but with red grape skins (Red Previpect), and three commercial prebiotics: inulin, grape pectin, and grape seed extract (GSPE).

## Materials and Methods

### Prebiotic Treatments

The new prebiotic was originated from the Grenache variety white class of *V. vinifera* L. according to the patent (Aldeguer et al., 2018). Red Previpect was produced using the same methodology but with Syrah red variety of *V. vinifera* L. Its performance was compared with the following commercial preparations of prebiotic: inulin and GSPE (The Hut Group, Cheshire, United Kingdom), and grapefruit pectin (Source Naturals, Santa Cruz, CA, United States). The fibre values were 89/100 g and 33/100 g for inulin and grape pectin, respectively.

### Compositional Analysis of Previpect

Chemical properties of Previpect are listed in Table 1. Moisture, ash, and total fat contents were determined by gravimetry. Protein content was determined by the Kjeldahl method volumetric assay (Anantakrishnan and Srinivasa Pai, 1952). The total carbohydrate content was calculated subtracting from 100 the sum of moisture, ash, proteins, total fat, and fibre. Calories were calculated according to the regulation 1169/2011 (Regulation (EU) No 1169/2011, 2011). Dietary and insoluble fibre were determined by gravimetry and enzymatic methods, from which soluble fibre was calculated. Calcium, phosphorus, magnesium, and potassium were measured by Inductively Coupled Plasma (ICP). The analysis was performed at Laboratorio LINAS (Maçanet de la Selva, Spain).

**TABLE 1:** Chemical properties of Previpect.

Main components	Average ± standard deviation (*n* = 2)
Moisture (%)	9.800.57
Ash (%)	4.540.42
Protein (%)	6.290.70
Total carbohydrate (%)	46.256.01
Total fat (%)	3.700.49
Dietary fibre (%)	30.303.68
Soluble fibre (%)	2.900.28
Insoluble fibre (%)	27.403.39
Calories (Kcal/100 g)	3049.90
Calcium (mg/Kg)	1913.5354.26
Phosphorus (mg/Kg)	1745247.49
Magnesium (mg/Kg)	62331.11
Potassium (mg/Kg)	152702390

### *In vitro* Human Digestion

Previpect is intended to be administered using gastro-resistant capsules. For this reason, all substrates, except Previpect (see Supplementary Table 1), were digested *in vitro* under appropriate conditions before being added into the faecal slurry, and following the procedures described by Maccaferri et al. (2012). In this experiment, digestion was not monitored by ion-exchange chromatography.

### Study Design

In this proof of concept, 15 fresh faeces were collected at Hospital Dr. Josep Trueta (Girona, Spain), nine of which were from patients with intestinal disorders (six IBD and three IBS) and six from CS (Table 2). CS were individuals without food intolerances, inflammatory bowel diseases, intestinal syndromes or neoplasms, and without clinical symptomatology. IBD patients presented clinical activity according to the Partial Mayo score (Lewis et al., 2008) for ulcerative colitis (UC, *N* = 3) and according to Harvey-Bradshaw index (Best, 2006) for Crohn’s disease (CD, *N* = 3), respectively. IBS patients were diagnosed according to Rome IV criteria. Participants followed regular diets and had not been treated with antibiotics, prebiotics, and/or probiotics for at least 1 month. Volunteers with severe comorbidities, pregnancy, previous surgeries that compromised the digestive system, or those who had received chemotherapy or radiation therapy in the past 6 months were excluded.

**TABLE 2:** Population characteristics.

	*n*	Age (mean, range)	Gender, female (%)	Clinical index
UC	3	59 (41–71)	100	≥5^†^
CD	3	50 (37–61)	100	≥5^‡^
IBS	3	45 (33–57)	33	–
CS	6	34 (29–39)	33	–

*UC, ulcerative colitis; CD, Crohn’s disease; IBS, irritable bowel syndrome; CS, control subjects.*

*^†^Partial Mayo Score.*

*^‡^Harvey-Bradshaw index.*

### Faecal Slurry Preparation and Incubation

The samples were collected in sterile containers and kept at room temperature for less than 4 h. Faecal samples were diluted 1:5 (w:v) with fermentation buffer (0.1 M KH_2_PO_4_, 0.05 mM NaOH; pH 7.0) (Waldecker et al., 2008) in a sterile plastic bag. The bags were carefully manually squeezed to mix the content.

All prebiotics (200 mg) were weighed in triplicates inside 20 ml screw-cap tubes with 10 ml of fermentation buffer previously degassed by increasing temperature to 100°C for 10 min. Faecal slurry (10 ml) was added to each tube, being the final concentration a 10% of the received stool sample. Tubes were tightly sealed and incubated at 37°C under gentle agitation (120 rpm) for 72 h. A blank without any fibre was used as a control of the *in vitro* fermentation process.

### DNA Extraction and Bacteria Quantification by Real-Time PCR

Once the incubation had finished, one aliquot from each fermentation triplicate was separated to be used for the DNA extraction. The total DNA was extracted from each fermentation aliquot using the NucleoSpin Soil DNA kit (Macherey-Nagel GmbH & Co., Düren, Germany), following the manufacturer’s instructions and eluting DNA in a final volume of 100 μL SE Elution buffer.

Abundance of 6 microbial markers were analysed by quantifications with real-time polymerase chain reactions (qPCR): *F. prausnitzii* (FPRA) and their two phylogroups (PHGI, PHGII), *Akkermansia muciniphila* (AKK), *Roseburia sp.* (ROS), and B46 (best BLAST match *Subdoligranulum variabile*).

Quantification of AKK, ROS, and B46 was performed by preparing single reactions of each biomarker using the different biomarkers was performed by preparing a single reaction for each biomarker using GoTaq qPCR Bryt Master mix (GoTaq^®^ qPCR Master Mix, Promega, Madison, WI, United States). Reactions consisted of 10 μl containing 1× GoTaq^®^ qPCR Master Mix (Promega) and between 12 and 20 ng of genomic DNA template. Quantification of FPRA, PHGI, and PHGII, was performed by preparing a single reaction for each biomarker using GoTaq qPCR Probe Master Mix (GoTaq^®^ qPCR Master Mix, Promega, Madison, WI, United States). Reactions consisted of 10 μL containing 1× GoTaq qPCR Master Mix (Promega) and between 12 and 20 ng of genomic DNA template. Primers used in this study were purchased from Macrogen (Macrogen, Seoul, South Korea).

The 16S rDNA-targetting primers and probes used in this study are shown in Table 3. Primers concentration for FPRA was 250 nM (300 nM for probe), for its two phylogroups 300 nM (probe 900 nM), AKK 250 nM, ROS 150 nM, and B46 300 nM. For all qPCR, an initial denaturation step was set at 95°C for 10 min and a total of 40 cycles were performed (Table 3). Samples were run in duplicate on the same plate. For data analysis, the mean of duplicate quantifications was used. RT-PCRs were performed with the AriaMx thermocycler (Agilent Technologies, Santa Clara, CA, United States). All samples were amplified in duplicate, which were considered valid when the difference between threshold cycles (Ct) was less than 0.6. A no template control reaction was included in each qPCR run.

**TABLE 3:** 16S rRNA-targetted primers and probes (when applicable) sequences used in this study with the RT-PCR conditions.

	Primers and probes			RT-PCR conditions for the 40 cycles	
		
Target	Primers	Sequence 5′-3′	References	Denaturing (°C; s)	Annealing and extension (°C; s)
*F. prausnitzii*	Fpra_F	TGTAAACTCCTGTTGTTGAGGAAGATAA	Lopez-Siles et al., 2014	95; 15	60; 60
	Fpra_R	GCGCTCCCTTTACACCCA			
	Fpra_PR	FAM-CAAGGAAGTGACGGCTAACTACGTGCCAG-TAMRA			
*F. prausnitzii*	PHG_F	CTCAAAGAGGGGGACAACAGTT	Lopez-Siles et al., 2016	95; 15	64; 60
phylogroup I and	PHG_R	GCCATCTCAAAGCGGATTG			
phylogroup II	PHGI_PR	FAM-TAAGCCCACGACCCGGCATCG-BHQ1			
	PHGII_PR	HEX-TAAGCCCACRGCTCGGCATC-BHQ1			
*A. muciniphila*	AKK_F	CAGCACGTGAAGGTGGGGAC	Collado et al., 2007	95; 15	60; 60
	AKK_R	CCTTGCGGTTGGCTTCAGAT			
*Roseburia* spp.	ROS_F	TACTGCATTGGAAACTGTCG	Larsen, 2019	95;15	60; 60
	ROS_R	CGGCACCGAAGAGCAAT			
B46	B46_F	GTACGGGGAGCAGCAGTG	Malagón et al., 2019	95; 15	62; 45
	B46_R	GACACTCTAGA GCACAGTTTCC			

### Short Chain Fatty Acids

After separating the aliquot used for the DNA extraction, the tubes were centrifuged for 30 min at 4500 × *g* at 4°C. The supernatants were transferred into new tubes and centrifuged again at 4500 × *g* and 4°C for 15 min. Supernatants from faecal incubations were sterilised by filtration using a pore size of 0.22 μmØ.

Acetate, propionate, and butyrate were analysed using a gas chromatograph (Agilent 7890A GC system, Agilent Technologies, Santa Clara, CA, United States) equipped with a fuse-silica capillary column (DB-FFAP, 30 m × 0.32 mm × 0.5 μm) and a flame ionization detector (FID). The analysis was performed at Research Technical Services from UdG (Girona, Spain). Crotonic acid was used as internal standard. 0.5 μL of each sample was injected in split mode at 275°C. The analyses were performed using the following temperature programme: 3 min at 40°C, 5°C min^–1^ to 70°C, 7°C min^–1^ to 120°C, 10°C min^–1^ to 180°C, and 35°C min^–1^ to 250°C, hold for 5 min. All the analysed compounds were previously identified and calibrated by using a bench of standard solutions. The standard solutions were prepared by diluting a stock solution of the primary compounds.

### Statistical Analysis

As stated before, all the experiments were conducted in triplicate. Goodness-of-fit of the bacterial population and SCFA data to normal distribution was tested using Shapiro–Wilk W test. Due to the non-normal distribution, the Mann–Whitney U test was performed to compare the measurements between two treatments using SPSS (version 23.0, Chicago, IL, United States). MANOVA (Wilks’ Lambda test) was performed with RStudio (version 3.5.0, Boston, MA, United States) after data conversion to geometric for the calculation of the geometrical mean of all the variables and for each diagnosis using CoDaPack (version 2.02.21, Girona, Spain). A *p*-value < 0.050 was considered statistically significant. Graphs were performed using GraphPad Prism 5.0 (GraphPad Software, La Jolla, CA, United States).

## Results

### Bacterial Markers

The effect on bacterial populations after the *in vitro* fermentation process that occurs in faecal samples supplemented with Previpect and the other prebiotics was determined using qPCR (Table 4).

**TABLE 4:** Mean Ct abundances and standard deviation of bacterial markers *F. prausnitzii* (Fpra), *A. muciniphila* (AKK), *Roseburia* spp. (ROS), and *S. variabile* (B46), and *F. prausnitzii* phylogroup I (PHGI) and phylogroup II (PHGII), for control subjects (CS, *N* = 6), irritable bowel syndrome (IBS, *N* = 3), ulcerative colitis (UC, *N* = 3), and Crohn’s disease (CD, *N* = 3) samples.

Inoculum	Substrate	Fpra	AKK	ROS	B46	PHG I	PHG II
CS	Negative control	14.651.21^a^	21.737.09^a^	18.591.94^a^	19.011.31^a^	16.781.50^a^	17.331.56^a^
	Previpect	13.420.59^b^	21.257.54^a^	15.212.44^b^	18.310.43^a^	15.671.46^b^	16.421.05^a^
	Red Previpect	14.551.36^a^	21.166.97^a^	18.612.09^a^	18.931.43^a^	16.541.48^a^	17.331.71^a^
	Inulin	14.021.24^a^	19.707.21^a^	18.593.21^a^	18.631.40^a^	15.961.51^a^	16.871.51^a^
	Grape pectin	14.181.36^a^	20.277.73^a^	18.823.16^a^	18.861.64^a^	16.892.36^a^	17.252.17^a^
	GSPE	14.171.21^a^	21.346.90^a^	16.321.01^b^	18.610.98^a^	16.992.33^a^	16.661.14^a^
IBS	Negative control	16.441.28^abc^	14.210.49^abc^	18.931.33^a^	20.420.76^abc^	18.190.87^ab^	19.751.58^ab^
	Previpect	15.611.19^b^	14.440.39^b^	17.731.49^b^	20.060.48^b^	17.720.82^a^	18.971.64^a^
	Red Previpect	16.630.83^c^	14.090.39^a^	19.460.99^a^	20.680.68^c^	18.480.53^a^	19.871.39^a^
	Inulin	15.931.27^a^	14.240.50^a^	19.530.45^a^	20.280.57^a^	18.021.02^bc^	19.301.76^bc^
	Grape pectin	15.361.23^a^	14.260.67^a^	19.491.09^a^	19.920.66^a^	17.310.92^c^	18.641.63^c^
	GSPE	15.621.42^a^	13.800.40^c^	17.541.72^b^	19.721.10^a^	17.511.16^a^	18.981.92^a^
UC	Negative control	17.160.90^a^	21.830.82^a^	20.2810.03^a^	21.421.86^a^	20.011.07^a^	20.400.58^a^
	Previpect	15.402.11^a^	24.879.53^a^	17.391.76^b^	21.814.56^a^	18.081.88^a^	21.016.57^a^
	Red Previpect	16.810.97^a^	23.7510.43^a^	19.331.70^ab^	21.151.30^a^	19.590.82^a^	20.191.86^a^
	Inulin	16.401.54^a^	23.8610.40^a^	20.321.96^a^	20.571.89^a^	18.611.66^ac^	19.632.31^a^
	Grape pectin	16.041.48^a^	24.929.84^a^	19.761.94^a^	20.441.85^a^	17.891.58^bc^	18.942.29^a^
	GSPE	16.471.32^a^	23.7110.54^a^	19.683.07^ab^	20.781.21^a^	19.100.96^ac^	19.631.89^a^
CD	Negative control	15.703.21^a^	14.442.35^ab^	17.251.94^ab^	20.223.08^abc^	19.383.13^a^	20.317.36^a^
	Previpect	14.342.69^a^	16.413.34^a^	16.051.43^a^	18.862.17^a^	18.143.58^a^	19.076.59^a^
	Red Previpect	14.611.45^a^	14.102.12^b^	18.071.30^b^	19.011.12^b^	17.652.44^a^	19.145.21^a^
	Inulin	14.811.06^a^	14.322.62^ab^	19.211.81^bc^	19.210.88^b^	18.743.92^a^	19.264.14^a^
	Grape pectin	15.901.27^a^	14.682.77^ab^	20.131.22^c^	20.451.04^c^	19.732.56^a^	20.704.83^a^
	GSPE	16.083.83^a^	15.304.27^ab^	17.452.23^b^	20.373.21^a^	19.413.29^a^	20.928.37^a^

*Statistical comparisons between groups are indicated by superscript letters: different letters indicate statistically significant differences (*p* < 0.05) between groups.*

**FIGURE 1:**
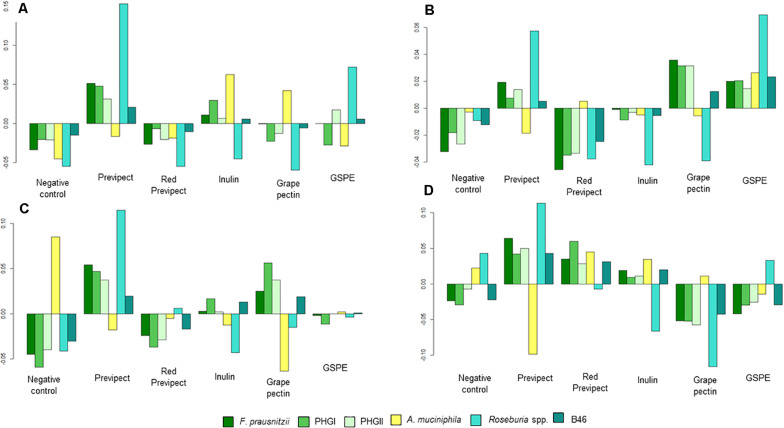
Geometric mean for each of the four population types: **(A)** control subjects, **(B)** irritable bowel syndrome, **(C)** ulcerative colitis, and **(D)** Crohn’s disease; compared with the overall mean of each substrate condition and bacterial marker.

Several significant differences were found when the abundance of the different analysed bacterial markers was compared in all the tested fibres, being Wilks test significant (*p*-value < 0.001) for all treatments (Figure 1). These results show that Previpect enabled the growth of all the analysed bacteria except AKK, whose abundance was decreased in all inocula. ROS was the bacterial marker with a most prominent increment in its abundance as a result of Previpect incubation, followed by both *F. prausnitzii* phylogroups and B46. In samples from CD patients, Previpect did not show any effect when compared to the negative control. Furthermore, in samples from CS, Previpect had a significant effect by boosting the abundance of FPRA and its phylogroup I (*p*-value = 0.001 and *p*-value = 0.019, respectively). Although the repercussion caused by fibres in the analysed bacteria changed according to the condition of sample donor, Previpect demonstrated stability in all of them.

Previpect induced higher increment in the abundance of the studied bacterial markers than red Previpect regarding ROS in CS (*p*-value = 0.003), CD (*p*-value = 0.019), and IBS samples (*p*-value = 0.015), as with FPRA in CS and B46 in IBS patients (*p*-value = 0.007 and *p*-value = 0.031, respectively). Previpect showed also significantly higher efficiency than inulin in stimulating the growth of ROS in all samples. Previpect showed similar results as inulin on the ability to increase the abundance of ROS. Grape pectin did not show significant differences when it was compared to Previpect in any inocula except for UC samples. Concerning GSPE, no differences were found in CD samples when compared to Previpect, although it was significantly better increasing AKK abundance in IBS samples (*p*-value = 0.012). However, Previpect significantly augmented FPRA abundance in CS (*p*-value = 0.032).

### Short Chain Fatty Acids

The fermentation of faecal slurry was carried out under standard conditions and led to the formation of the SCFAs acetate, propionate, and butyrate. Total SCFA, which is the sum of acetate, propionate, and butyrate, is used as an indicator of fibre fermentability (Jonathan et al., 2012). Concentrations of SCFA after the *in vitro* fermentation experiment are presented in Table 5. The prebiotic fermentation cultures contained significantly higher concentrations of SCFA than the blank for all substrate conditions and for all inocula (*p* < 0.001). Previpect produced from its fermentation the highest concentration of total SCFA in all inocula, becoming the most suitable substrate for SCFA production. The lowest total SCFA yield was the one obtained from GSPE fibre, except for UC inocula, in which the lowest amount of total SCFA was produced from Red Previpect.

**TABLE 5:** Butyrate, acetate, propionate, and total short chain fatty acids (SCFA) concentration (mg/L) after the *in vitro* fermentation of Previpect, red Previpect, inulin, grape pectin, and grape seed extract (GSPE) using faeces from control subject (*n* = 6), irritable bowel syndrome (*n* = 3), ulcerative colitis (*n* = 3), and Crohn’s disease patients (*n* = 3) as inocula.

	Control subjects inoculum				Irritable bowel syndrome inoculum			
		
Condition	Butyrate (mg/L)	Acetate (mg/L)	Propionate (mg/L)	Total SCFA (mg/L)	Butyrate (mg/L)	Acetate (mg/L)	Propionate (mg/L)	Total SCFA (mg/L)
Negative control	308.84113.32***	573.13131.54***	291.56135.91***	424.41333.62***	293.33193.07***	648.05353.88***	260.05241.08***	1201.43699.48***
Previpect	1046.76405.03	2162.87648.14	852.65294.74	4062.281147.52	767.75301.21	2376.85198.45	858.1650.08	4002.76497.45
Red Previpect	363.51165.90***	771.98159.42***	372.74167.04***	1508.23400.89***	385.26172.43*	1058.91568.44***	407.45158.61***	1851.61814.63***
Inulin	758.78329.93*	1399.43500.93***	609.49275.89**	2767.701020.29***	611.29261.72	1585.68528.91***	637.40264.57	2834.37981.67**
Grape pectin	823.34426.60	1840.93663.95	736.47322.46	3400.741222.30*	686.42255.15	2230.85305.34	818.42149.81	3735.69426.41
GSPE	358.20236.40***	696.37311.22***	337.19255.69***	1391.75787.56***	328.10159.95*	1098.00517.74***	339.85174.56***	1765.94785.88***

	**Ulcerative colitis inoculum**				**Crohn’s disease inoculum**			
		
**Condition**	**Butyrate (mg/L)**	**Acetate (mg/L)**	**Propionate (mg/L)**	**Total SCFA (mg/L)**	**Butyrate (mg/L)**	**Acetate (mg/L)**	**Propionate (mg/L)**	**Total SCFA (mg/L)**

Negative control	321.3072.58***	581.18109.64***	359.5744.54***	1262.05196.05***	409.56161.82***	672.40384.30***	410.17126.48***	1492.13663.08***
Previpect	759.11130.34	1777.52322.23	1072.37371.19	3609.00724.51	1277.54442.09	2119.07451.17	860.10209.95	4256.71995.58
Red Previpect	354.44135.35***	725.15194.70***	401.6788.63***	1481.26398.82***	564.43189.43**	1000.37326.71***	550.92128.02**	2115.72622.36***
Inulin	479.00124.67**	1233.69448.17*	773.09370.06	2485.78924.24*	769.58334.83*	1168.70540.70**	630.37208.31*	2568.651069.66**
Grape pectin	556.51151.98*	1465.65286.63*	709.42122.62*	2731.58495.27*	803.03389.87	1644.72566.68	752.09169.01	3199.831078.88
GSPE	392.7575.89***	809.92221.67***	426.75153.07***	1629.42426.89***	404.82168.37***	742.60300.13***	365.31132.14***	1512.73***

Significant differences between fibres and Previpect are shown as *p-value < 0.05, **p-value ≤ 0.01, ***p-value ≤ 0.001.

**FIGURE 2:**
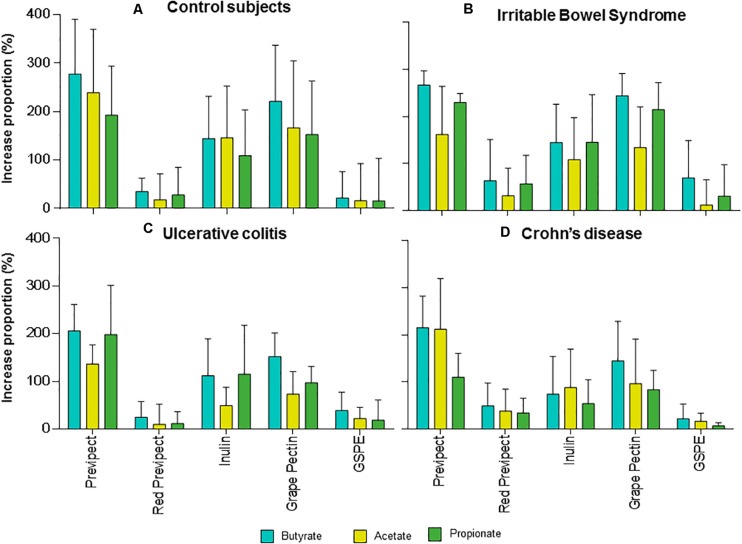
Mean and standard errors of the increase proportions (%) of the short-chain fatty acids: acetate (blue), butyrate (yellow), and propionate (green) regarding negative control of the process produced during the *in vitro* fermentation for Previpect, red Previpect, inulin, grape pectin, and GSPE fibres using faecal inocula from **(A)** control subjects, **(B)** irritable bowel syndrome, **(C)** ulcerative colitis, and **(D)** Crohn’s disease patients.

Acetate was the most abundant SCFA derived from Previpect fermentation (Figure 2), which increased between 205.85 and 277.38% the amount produced by the blank, followed by butyrate (136.26–238.94% increase), and finally, propionate, whose yielding was comprised between 109.69 and 230.00% among inocula. Total SCFA concentrations were increased 1.8-fold with respect to those of negative control in both UC and CD samples means (*p*-value = 0.001 and *p*-value < 0.001, respectively), reaching 2.3-fold increase in IBS and the highest increase in CS, being 2.5-fold (*p*-value = 0.001).

No significant differences were observed in the production of SCFA when Previpect was compared to grape pectin in CS, IBS, and CD samples. Nevertheless, Previpect showed higher SCFA production than grape pectin in UC samples. Besides, Previpect performed significantly better than inulin in CS and CD samples but showed no significant differences in propionate concentration in UC samples. Previpect was also superior enhancing acetate and butyrate production. In IBS inocula, inulin showed no significant differences concerning butyrate and propionate. However, Previpect was better at inducing in acetate production. Previpect fermentability resulted in significantly higher levels of SCFA than red Previpect and GSPE substrates.

## Discussion

Annually the processing of grapes (*V. vinifera* L.) for wine production leaves behind 14.5 million tons of grape by-products from wineries or “grape pomace” result in Europe. This pomace mainly consists of the fruit skins and seeds, as it results from the pressing of the fruit (Arnous and Meyer, 2008). The skins of grapes are known to be rich sources of phenolic compounds (Pinelo et al., 2006). Grape skins represent about 5–10% of the total dry weight of the grape and are generally treated as a waste product, despite containing an array of flavonoids, polyphenols, and anthocyanins. These molecules have been shown to produce health benefits associated with antioxidant, cardioprotective, hepatoprotective, anticarcinogenic, and antidiabetic effects, among others (Nassiri-asl and Hosseinzadeh, 2009). Grape skins also contain considerable amounts of potential prebiotic indigestible carbohydrates made up of 30% neutral polysaccharides (cellulose, xyloglucan, arabinan, galactan, xylan, and mannan), 20% of acidic pectin substances (62% of which are methyl esterified), ∼15% insoluble proanthocyanidins, and <5% structural proteins (Pinelo et al., 2006). In addition to all of the aforementioned features, this study has shown that Previpect is also capable of enhancing the growth or metabolic activity of some beneficial bacterial species of the gut microbiota.

The analysed species were chosen because of the importance of their function in the large bowel health. *F. prausnitzii* (together with its two phylogroups) is one of the leading components of the microbiota and the most recognisable butyrate-producing bacteria in the human colon (Sousa et al., 2017); *S. variabile* is a butyrate producer closely related to *F. prausnitzii* (Holmstrøm and Lawson, 2015); *Roseburia* spp. comprises different butyrate-producing bacteria, which also produce propionate, such as *R*oseburia *inulinivorans*; and *A. muciniphila* is an acetate producing bacteria (Koh et al., 2016). The results of this study demonstrate the great fermentative ability of Previpect, enabling the growth of specially *Roseburia* spp., but also that of *F. prausnitzii*, its two phylogroups and *S. variabile* in all inocula. This fermentative potential has been reflected in a considerable increase in the three SCFA analysed (acetic, propionic, and butyric acid), produced not only by the quantified markers but also by all the SCFA-producing bacteria found in the intestinal microbiota of the analysed samples, increasing their concentration up to 2.5-fold. Acetic, propionic, and butyric acids are key microbial fermentation products that are known to be beneficial for human health. Acetate is the most prominent SCFA and substrate for butyrate production reported to have effects on lipid metabolism, such as lipogenesis and cholesterogenesis (Morrison et al., 2016). Propionic acid regulates glucose homoeostasis in the liver (Besten et al., 2013). Lower faecal concentrations of acetate and propionate have been observed in UC samples (Machiels et al., 2014). Butyric acid plays a crucial role in maintaining human gut health, as is the primary energy source for colonocytes (Cummings, 1981), a regulator of gene expression, immune cell growth, and apoptosis in host cells (Vinolo et al., 2011), and protects against colitis and colonic cancer (Cook and Sellin, 1998). Therefore, butyrate prevents mucosal atrophy by improving the mucosal barrier function and exhibits immunomodulatory effects and anti-inflammatory properties. Finally, butyrate has a strong effect on IBD through improving the mucosal layer and inhibiting inflammation (Russo et al., 2019). Observations described in all these studies suggest that Previpect may enhance a healthy state of human intestine.

As for IBS patients, since pathogenesis is a matter of scientific debate, treatment focuses on the relief of symptoms such as bloating, abdominal pain, diarrhoea, and constipation. The fact that the passive absorption of water in the colon depends on the presence of SCFAs may explain the potential role of butyrate in clinical conditions involving diarrhoea due to propionate decreases colon motility (Ríos-Covián et al., 2016). The relief of abdominal pain is an essential aspect of IBS treatment. Butyrate has a probable beneficial influence on the hypersensitivity of intestinal receptors, which results in a decrease of intraintestinal pressure by improving bowel peristalsis and retractability of the circular muscle layer (Załęski et al., 2013; Farup et al., 2016; Koh et al., 2016).

Several investigations have shown that certain butyrate-producing Firmicutes bacteria are reduced in IBD. In particular, numbers of *F. prausnitzii* in faecal and gut mucosa samples are reduced in CD and UC (Sokol et al., 2006, 2009; Lopez-Siles et al., 2014, 2016). *Roseburia* spp. and *A. muciniphila* are also depleted in IBD mucosa and faecal samples from UC patients (Derrien et al., 2017).

Inulin is a natural component in several foods such as leek, asparagus, chicory, Jerusalem artichoke, garlic, artichoke, onion, wheat, banana, and oats (Gibson, 1999). The prebiotic activity of inulin-type fructans has been extensively confirmed. These prebiotics target microorganisms like bifidobacteria, which significantly increase in number after ingestion (Kolida et al., 2002; Kolida and Gibson, 2007), and are the current market leaders. Concerning *F. prausnitzii* strains, they have not demonstrated the ability to ferment inulin (Lopez-Siles et al., 2017) whereas Previpect has proved it.

Pectin is considered a soluble dietary fibre and exerts physiological effects on the gastrointestinal tract, such as reducing glucose absorption, enhancing hypocholesterolemia effect, and delaying gastric emptying. Pectin is found in sugar beet pulp, peach peels, pulps of grapes, and pumpkin or apples, which has also demonstrated the ability to stimulate the growth of *Bifidobacterium* (Gómez et al., 2016; Ho et al., 2017). Interestingly, most of *F. prausnitzii* strains grow on apple pectin, although not on citrus pectin (Lopez-Siles et al., 2012), revealing that not all pectins serve to stimulate their growth. In this study it has been reflected that *F. prausnitzii* grows with grape pectin and that similar prebiotic effects are obtained compared to Previpect since no significant differences were observed.

Previous studies have revealed several health beneficial effects of wine grape seed flour or extract (GSF or GSPE), a by-product of winemaking, such as hypolipidemic and anti-obesity properties attributed to high contents of flavonoids (Lee et al., 2018). Health beneficial effects of GSPE are closely associated with modulation of the intestinal microbiota, mainly producing a prebiotic effect on *Akkermansia* sp. (Anhê et al., 2015), confirmed by our results and unlike Previpect, in which *A. muciniphila* was the only microorganism analysed presenting a significant decrease in inocula.

Since flavonoids are more abundant in red than white grapes, the effect of the Previpect was compared with a Previpect made from a red class of grapes, to see if its effect was distinct and dependent on flavonoids (Anhê et al., 2015). Thus, the ability exhibited by Previpect goes beyond to the flavonoids since it presented a higher capacity to increase the abundances of beneficial anaerobic bacteria species and to enhance the production of SCFA than Red Previpect.

All these comparisons with commercial prebiotics lead us to conclude that Previpect is considerably similar to grape pectin, and, therefore, Previpect must have a high content of this compound. Nonetheless, Previpect is significantly superior to pectin in stimulating the growth of *Roseburia*, and in the absence of bibliographic references in this regard, it cannot be determined which compound confers this ability.

Despite these promising results, we readily acknowledge that a more robust examination in larger cohorts is essential prior to commercial application, as well as, clinical studies *in vivo*.

## Conclusion

In conclusion, Previpect supplementation seems to be a promising tool for IBD and IBS treatment strategies, but also for CS in order to sustain a good gut health and as a preventive measure for temporary dysbiosis.

## Data Availability Statement

The datasets presented in this study can be found in online repositories. The names of the repository/repositories and accession number(s) can be found in the article/Supplementary Material.

## Ethics Statement

The studies involving human participants were reviewed and approved by the Comitè d’Ètica d’Investigació Clínica de Girona. The patients/participants provided their written informed consent to participate in this study.

## Author Contributions

LO: methodology, formal analysis, investigation, resources, writing – original draft, and visualisation. SR-P: methodology, formal analysis, investigation, writing – review and editing, and visualisation. JA, MM, and MS: investigation. AB, AL, LT, DB, and LP: resources. MS-P, XA, and JG-G: conceptualisation, methodology, formal analysis, writing – review and editing, and visualisation. All authors contributed important intellectual content during manuscript drafting or revision and approved the final draft.

## Conflict of Interest

LO, SR-P, JA, MM, MS, MS-P, and XA were employed by company GoodGut SL, Girona, Spain. The remaining authors declare that the research was conducted in the absence of any commercial or financial relationships that could be construed as a potential conflict of interest.
