# Supplementary material for: A Novel Grape-Derived Prebiotic Selectively Enhances Abundance and Metabolic Activity of Butyrate-Producing Bacteria in Faecal Samples
Source: Front Microbiol. 2021 Mar 23;12:639948. doi: 10.3389/fmicb.2021.639948 (PMC8021714; doi:10.3389/fmicb.2021.639948)
Supplement: Supplementary file 1 [file Table_1.DOCX]

**SUPPLEMENTARY INFORMATION**

*In vitro* digested Previpect lost much of its ability to stimulate the growth of butyrate-producing bacteria when compared with the values ​​obtained with the undigested Previpect, obtaining in most cases a result similar to the negative control.

**Table S1.** Mean Ct abundances (N=2) of bacterial markers (*S. variabile* (B46), *F. prausnitzii* (Fpra), and its phylogroup I (PHGI) and phylogroup II (PHGII), *Roseburia* spp. (ROS) and *A. muciniphila* (AKK)).

| **Condition** | **B46** | **Fpra** | **PHGI** | **PHGII** | **ROS** | **Akk** |
| --- | --- | --- | --- | --- | --- | --- |
| Negative control | 20.26 | 16.04 | 15.94 | 20.06 | 17.17 | 17.01 |
| Undigested Previpect | 18.37 | 13.72 | 12.96 | 18.91 | 15.09 | 17.36 |
| Digested Previpect | 20.15 | 16.23 | 15.60 | 20.03 | 17.79 | 16.44 |
